# Parent training tailored for parents with ADHD: a randomized controlled trial

**DOI:** 10.1186/s12888-025-07166-8

**Published:** 2025-08-26

**Authors:** Therese Lindström, Sofia Buddgård, Lena Westholm, Martin Forster, Sven Bölte, Tatja Hirvikoski

**Affiliations:** 1 Center of Neurodevelopmental Disorders (KIND), Department of Women’s and Children’s Health, Centre for Psychiatry Research, Karolinska Institutet and Region Stockholm, Stockholm, Sweden; 2 Habilitation and Health, Stockholm Health Care Services, Region Stockholm, Stockholm, Sweden; 3 Department of Clinical Neuroscience, Karolinska Institutet, Stockholm, Sweden; 4 Child and Adolescent Psychiatry, Stockholm Health Care Services, Region Stockholm, Stockholm, Sweden; 5 Curtin Autism Research Group, Curtin School of Allied Health, Curtin University, Perth, WA Australia

**Keywords:** Parental ADHD, ADHD adaptation, Parenting intervention efficacy, Parent training intervention, Externalizing behaviors

## Abstract

**Background:**

Parents who themselves have Attention-Deficit/Hyperactivity Disorder (ADHD) tend to benefit less from conventional parent training (PT) interventions than parents without ADHD, reporting suboptimal effects on both parenting-related outcomes and child externalizing or oppositional behaviors. Therefore, we examined the efficacy of a PT protocol tailored to adults with ADHD called Improving Parenting Skills Adult ADHD (IPSA), using a randomized controlled trial design.

**Methods:**

*N* = 109 self-referred parents with ADHD who had a child with or without ADHD aged 3 to 11 years were randomized to receive IPSA in addition to their routine services (*n* = 55) or to a comparison group continuing their routine services only (*n* = 54). Parent-reports of parental self-efficacy (primary outcome), parental stress, home chaos, and externalizing child behaviors (secondary outcomes) were collected pre and post IPSA, as well as at follow-up one and a half to three months later. The primary analyses were conducted according to the intention-to-treat principle, using linear mixed-effects models.

**Results:**

There was a larger pre-to-post increase in parental self-efficacy following IPSA compared to routine services only (Cohen’s *d* = 0.85, *p* < .001), which remained at follow-up (*d* = 0.84, *p* < .001). In addition, we observed a pattern of pre-to-post change in the expected direction across the study’s secondary outcomes (post intervention *d =* -0.39 to -0.71), including reductions in parental ratings of child externalizing behaviors. Forty-seven (96%) of the 49 parents who started IPSA completed the program, without signs of unintended harm.

**Conclusions:**

The IPSA PT program effectively and safely supported parents with ADHD in improving their parental self-efficacy. However, the long-term stability of the program’s effect requires further investigation, as does its effectiveness in a regular health care or social services context.

**Trial registration:**

The study was retrospectively registered during data collection, before analyses (clinicaltrials.gov, ID NCT06040996, 28/08/2023).

**Supplementary Information:**

The online version contains supplementary material available at 10.1186/s12888-025-07166-8.

## Introduction

Many of the tasks and activities typically associated with parenting make high demands on parents’ executive and self-regulation functions and can pose particular challenges for adults with Attention-Deficit/Hyperactivity Disorder (ADHD [1]). As indicated by reports of low parental self-efficacy [2] and high parental stress [3], it appears that many parents who themselves have ADHD experience an imbalance, or mismatch, between the demands of the parenting role and their parenting skills or other resources for coping with these demands. For example, it is common for parents with ADHD to report an increased reliance on ineffective, inconsistent, or even harsh parenting behaviors [4]. And given the familial nature of ADHD, many parents with ADHD will have children with ADHD traits and a predisposition to develop non-compliant behaviors that cause extra caregiving complexities [5–7]. Meanwhile, their day-to-day family life is often further complicated by household disorganization (e.g., home chaos [8]), co-occurring psychiatric conditions (e.g., depression [9]), and psychosocial challenge (e.g., socioeconomic disadvantage [10]). Taken together, it is essential that parents with ADHD can access parenting support when needed; preferably at an early stage, whether or not their child or children have already developed ADHD traits or externalizing behaviors.

Parents’ confidence in their own ability to bring-up their children competently and to exert a positive influence on their child’s development – that is, their parental self-efficacy – has been repeatedly linked to parenting efficacy, family adjustment, child well-being, and parental mental health [11–13]. Self-efficacy is key to behavior change including the initiation of challenging tasks [14, 15] and the construct has been identified as a therapy-relevant factor in interventions for adults with ADHD [16]. Relatedly, *parental* self-efficacy is an important target for support aimed at parents trying to develop their parenting while dealing with challenging, stressful, or otherwise unfavorable circumstances [12, 13, 17].

Behavioral parent training (BPT) is effective in helping parents improve their parental self-efficacy [17, 18] and develop parenting skills to prevent, manage, or reduce externalizing behaviors in their offspring, with or without ADHD [19, 20]. However, the success of BPT varies between families. Previous studies indicate that evidence-based BPT tends to produce less favorable outcomes in families where the parent has ADHD [21], with suboptimal effects seen for both parenting and child behaviors. Why this is the case needs to be further investigated, but many point to the fact that BPT– just like parenting– tends to put high demands on parents’ attentional, executive and self-regulatory functions [1, 21, 22]. Similarly, others have concluded that providing BPT without adequately considering parent characteristics (e.g., functioning profiles) and potential treatment barriers (e.g., a mismatch between BPT demands and resources available to the parent) might well lead to suboptimal or even adverse BPT outcomes [23–25].

Of note, there is a shortage of clinical trials of BPT involving parents meeting ADHD diagnostic criteria. One exception is a trial which found that eight weeks of individual BPT had more consistent effects on parenting than pharmacological treatment of the parents’ ADHD, although the authors also concluded that for most families, neither intervention was sufficient to significantly improve child functioning [26].

To date, most attempts to support parents with ADHD have focused on the possibility of treating the parents’ ADHD symptoms with pharmacological or non-pharmacological interventions (e.g., [27, 28]), rather than on adjusting the contents and delivery of BPT specifically for adults with ADHD. However, pharmacological treatment of parents’ ADHD seems to have limited effect on parenting and family functioning [21, 26]. And in a study randomizing mothers to multimodal treatment of adult ADHD or supportive counselling prior to BPT, treating mothers’ ADHD was associated with improvements in their ADHD symptoms, but not with enhanced BPT outcomes [28, 29]. Common to studies that have evaluated BPT in families where both child and parent has ADHD, is the use of individually delivered BPT, tailored to each family’s needs [26, 28, 30]. But so far, few studies have investigated the potential of a BPT protocol that has itself been adjusted to meet the needs of adults with ADHD [31].

*Improving Parenting Skills Adult ADHD* (IPSA) is a new BPT program tailored to parents with ADHD. It was developed using an iterative co-creation approach (for details, see [31]) resulting in a program that uses evidence-based BPT protocols [32, 33] but adapts content and delivery to increase relevance, accessibility, and implementability for parents who themselves have ADHD. Unlike more traditional BPT, IPSA combines structured group sessions with individual support by an occupational therapist (OT) who can help parents try out personalized strategies and tools to enable and enhance the execution of targeted tasks, activities, and skills at home [34]. In doing so, it draws on the benefits of both group-based and individual BPT - offering opportunities to share experiences and advice with peers, while providing individually tailored support to facilitate the implementation of BPT contents and skills. Additional measures to reduce common BPT barriers include the use of individualized appointment reminders and adaptations to the program materials, the approach of the BPT therapists, and the physical BPT environment. The clinical feasibility (e.g., acceptability, accessibility, and safety) of IPSA has been previously shown in an uncontrolled study [31], while its potential efficacy remains to be investigated.

In sum, although parents with ADHD appear to be at increased risk for both parenting challenges and suboptimal outcomes following conventional BPT, there is a lack of studies evaluating the potential of BPT tailored specifically to adults with ADHD. Thus, we conducted a randomized controlled trial (RCT) of the IPSA BPT program for adults with ADHD, to examine if participation in IPSA is associated with improvements in parental self-efficacy (primary outcome), parental stress, home chaos, or externalizing behaviors in the participant’s child (secondary outcomes). In addition, we assessed program completion rates and parent use of parenting skills and behaviors addressed by the intervention.

## Methods

### Study design and setting

A two-arm RCT with parallel groups was conducted to evaluate the efficacy of the IPSA BPT program delivered in addition to the participants’ routine services, compared with continued routine services only. Assessments using self- and parent-report scales were conducted at three time points: at baseline (pre), immediately after IPSA (post), and at follow-up (one and a half to three months after IPSA completion, in connection with an IPSA follow-up session). The study was retrospectively registered with ClinicalTrials.gov during data collection but before analyses (ID NCT06040996, 28/08/2023) and compiled using guidelines for reporting trials and describing interventions [35, 36]. It was carried out in cooperation with a publicly funded outpatient clinic offering group-based and psychoeducational interventions for families of children with ADHD (the ADHD Center, Habilitation & Health, Region Stockholm, Sweden). The clinic provided, among other things, clinical healthcare infrastructure, as well as the possibility to recruit participants among families enrolled at the clinic. Data was collected from January 2019 to January 2024.

### Participants

The sample consisted of self-referred parents with ADHD in need of parenting support, recruited via the project website and among families enrolled at the clinic involved. To be eligible for participation, parents needed to have an ADHD diagnosis (any presentation), at least one child aged 3 to 11 years with or without ADHD, and sufficient Swedish language proficiency to understand written material and rating scales. Their ADHD diagnoses, established within regular healthcare^1^, were corroborated as part of the eligibility assessment, by healthcare professionals (e.g., lic. psychologists or psychologists in training) who obtained access to assessment reports or equivalent diagnostic documentation (in ≥ 80%, ADHD combined presentation according to ICD-10 criteria). The study’s exclusion criteria included diagnosed autism or intellectual disability, as well as any severe psychiatric conditions (e.g., suicidality, psychosis, or substance use disorder) or crisis in the family that would hinder PT participation, as assessed by or in consultation with a study psychologist or OT. None of the participants were members of the same family.

### Procedure

Parents were assessed for eligibility in a structured telephone screening interview developed for the study (Suppl. Table 1), mapping family needs for support and interventions. Potentially eligible parents were invited to a complementary clinical assessment. They received both written and oral information about the study and its procedures before giving their written informed consent. The baseline assessment included questions about family characteristics, ongoing treatments, and measures of outcomes as well as safety. Eligible participants were randomly assigned (details below) to receive IPSA (treatment group) or to continue their routine services (comparison group) pending IPSA the following semester. The intervention was delivered by an OT and a psychologist, both with long (> 20 years) clinical experience and co-authors of the current study. For parents participating during the Covid-19 pandemic (two blocks, *n* = 36), most or all of IPSA had to be administered digitally, through available video services.

### Randomization

Eligible participants were block randomized (*n* = 18 at a time; ratio 1:1) to the treatment group (i.e., IPSA) or the comparison group (i.e., continued routine services). The random allocation sequences were generated by the study’s principal investigator, or a fellow doctoral student not otherwise involved in the current project, using a digital randomization tool (randomizer.org). The sequence and the allocation of individual participants were concealed (kept in opaque envelopes) from both participants and study staff administering the eligibility assessment and baseline questionnaires, until after the baseline assessment was completed.

### Sample size

Based on previous literature (e.g [30])., and initial evaluations of IPSA [31], we expected a medium effect size (ES, Cohen’s *d*) on the primary outcome. A priori power calculations (using an online power calculator) aiming for a power of 80% to detect medium ES (*d* = 0.50) at significance level alpha = 0.05 resulted in an estimated (intended) sample size of *n* ≥ 100 (in practice meaning six blocks of *n* = 18, to fill IPSA groups of *n* = 9).

### Interventions

#### IPSA

IPSA [31] is a BPT program for adults with ADHD who experience parenting difficulties and have at least one child with or without ADHD aged 3 to 11 years. It applies evidence-based BPT protocols [32, 33] but incorporates additional elements and adaptations to increase relevance and accessibility for adults with ADHD. The 14-session program is delivered in groups of nine parents by two group leaders (one OT and one psychologist or person with similar training) and followed by a booster group session. It alternates between group-based BPT (six sessions) and individual BPT support (eight sessions), such that bi-weekly group sessions (150 min) introducing new BPT skills are alternated with individual sessions (60 min, in the weeks in between) to facilitate their implementation. The six group sessions cover topics and skills such as: (i) how adult (parental) ADHD can manifest in and affect parenting and family life; (ii) how to strengthen one’s own (parental) prerequisites for managing challenging parent-child interaction situations; (iii) how to use positive reinforcement and build better parent-child relationships; (iv) how to communicate effectively and facilitate parent-child cooperation; (v, vi) how to use strategies to regulate one’s own (parental) emotional expressions in parent-child interactions and reduce the risk of parent-child conflict (Suppl. Table 2 provides a program outline). The majority of the individual support is offered by the OT and can be used flexibly for example to help identify BPT barriers, structure situations for BPT skills practice, address parents’ organizational and time management skills, and strengthen parents’ prerequisites for managing particularly challenging parent-child interaction situations. To further facilitate active BPT participation, individualized appointment reminders, inclusive language, information videos, cognitive aids, and visual supports are employed (for more information about the ‘ADHD adaptations’ made, see [31]). Treatment integrity is facilitated by a structured manual. Group sessions follow slide presentations including short lecture segments, video-based skill demonstrations, and information processing components such as discussions. Throughout their participation, parents can access BPT materials (e.g., summaries and information videos) online. Participants focus their BPT work and home assignments on *one* child, their IPSA ‘target child’.

##### Treatment fidelity

Treatment fidelity was assessed in a random subsample of 20% of group sessions held face-to-face. The assessment used audio recordings and checklists adapted from applicable intervention adherence instruments focusing on the implementation of key contents and components (yes or no; [37–39]. The assessment was made by research assistants (psychology students) who - after training by the first author and a quality assessment - made independent ratings. The percentage of content adherence averaged 94% (varying between 89% and 100%) across assessed sessions.

#### Continued routine services

All families continued their regular treatment plans and services throughout their study participation - either in parallel with IPSA (applies to the treatment group) or pending IPSA the following semester (applies to the comparison group). Details on service use in the treatment and comparison groups are presented in the results section. But in brief, most parents had at least one ongoing routine intervention, such as pharmacotherapy, psychological treatment, or psychosocial support (e.g., housing support).

### Study instruments

Parents completed primary and secondary outcome measures pre and post intervention, as well as at follow-up. At baseline, parents also rated their recent ADHD symptoms, using the Adult ADHD Self-Report Scale (ASRS) screener [40]. For all scales used, details on items, response scales, minimum/maximum scores, interpretation of sum scores, and internal consistencies (i.e., estimates of Cronbach’s alpha, a) are summarized in Table 1.

**Table 1:** Information on response scales, score ranges, interpretations, and internal consistencies of the Parent-Report questionnaires used

	Response scale	Items (*n*)	Min-max scores	Interpretation (better rating)^a^	α^b^
Primary outcome					
PSE Total Scale	0 (completely disagree) - 10 (totally agree)	48	0-480	Higher	.90
s1 Positive emotions		6	0-60	Higher	.65
s2 Being w/ your child		6	0-60	Higher	.82
s3 Empathy		6	0-60	Higher	.76
s4 Guiding		6	0-60	Higher	.72
s5 Rules		6	0-60	Higher	.78
s6 Pressures		6	0-60	Higher	.76
s7 Acceptance		6	0-60	Higher	.77
s8 Experience		6	0-60	Higher	.76
Secondary outcomes					
PSS	1 (strongly disagree) - 5 (strongly agree)	18	18-90	Lower	.84
CHAOS	1 (not at all) - 4 (very much)	15	15-60	Lower	.83
ECBI IS	1 (never) - 7 (always)	36	36-252	Lower	.91
ECBI PS	0 (no) or 1 (yes)	36	0-36	Lower	.86
Safety measures					
PSS-10	0 (never) - 4 (very often)	10	0-40	Lower	.81
HADS Anxiety	0 - 3 (anchors vary)	7	0-21	Lower	.83
HADS Depression	0 - 3 (anchors vary)	7	0-21	Lower	.83
Other measures					
ASRS screener	0 (never) - 4 (very often)	6	0-6	Lower	.66
Skills use	0 (never) - 6 (very often)	11	0-66	Higher	.66

*ASRS* Adult ADHD Self-Report Scale, *CHAOS* Confusion, Hubbub, and Order Scale, *ECBI IS* Eyberg Child Behavior Inventory, Intensity Scale, *ECBI PS* ECBI Problem Scale, *HADS* Hospital Anxiety and Depression Scale, *PSE* Parental Self-Efficacy scale, *PSS* Parental Stress Scale, *PSS-10* Perceived Stress Scale, 10-item version, *s1-sX* subscale 1-subscale X

^a^States whether a higher or a lower score is “better”, that is, which type of score (a higher or a lower) indicates the desired or preferred level of the rated construct, or in the case of the ASRS screener, a lower symptom rating

^b^Internal consistency estimated as *α *(Cronbach’s alpha)

#### Primary outcome measure

The Parental Self-Efficacy (PSE) scale (based on [41], adapted for a Swedish context by [42]) assesses parental self-efficacy across eight parenting domains, each constituting a subscale: positive emotions (e.g., *I can show my child affection*), being with your child (e.g., *I can plan things that my child enjoys doing*), empathy (e.g., *I understand my child’s needs*), guiding (e.g., *I remain calm when my child misbehaves*), rules (e.g., *I can stick to the rules I have set for my child*), pressures (e.g., *I have difficulty managing other people’s expectations of me as a parent*, reversed coded), acceptance (e.g., *I know that I am a good enough parent*) and experience (e.g., *I can make the changes needed to improve my child’s behavior*). The PSE Total Scale (summing all 48 items; details in Table 1) was used as primary outcome, while the eight PSE subscales (6 items each) were treated as secondary. The internal consistency of the Total Scale has previously been estimated at a = 0.94 in a Swedish sample [42]. In the current trial, it was a = 0.90 at baseline, a = 0.92 post intervention, a = 0.94 at follow-up. For more details, see Table 1.

#### Secondary outcome measures

The Parental Stress Scale (PSS [43]) was used to assess parental stress, including parents’ perceptions of parental stressors and distress as well as a potential lack of parental rewards or satisfaction [44]. The Confusion, Hubbub, and Order Scale (CHAOS [45]) was used to assess levels of home chaos, that is, the degree to which parents perceive their family home environment as disorganized, chaotic, cluttered, or hurried. The Eyberg Child Behavior Inventory (ECBI [46, 47]) was used to assess the occurrence and parental perceptions of externalizing behaviors in the participants’ IPSA target children. It consists of two dimensions: the Intensity Scale (ECBI IS) that measures the frequency of 36 child behaviors (e.g., defiance, non-compliance, and aggressiveness) and the Problem Scale (ECBI PS) which asks whether the targeted behaviors are perceived as problematic or not (yes or no). For details, see Table 1.

#### Measures to detect unintended harm

The 10-item Perceived Stress Scale (PSS-10 [48, 49]) and the Hospital Anxiety and Depression Scale (HADS; [50, 51]) were used to detect any deterioration with regard to parental mental health. Spontaneously reported adverse events (e.g., any unfavorable, potentially negative, event that occurred during the study period) and serious adverse events (e.g., threatening life or function, requiring hospitalization) were documented and reviewed (IPSA participants only). For details, see Table 1.

#### Assessment of program completion and use of targeted skills

Program completers were defined as program starters (i.e., parents attending at least one IPSA session) who attended at least nine (≈ two-thirds) of the 14 IPSA sessions. Participants’ use of BPT skills targeted during the intervention was assessed with 11 items on parenting behaviors (e.g., having moments of parent-child quality time, using positive reinforcement, or managing to behave calmly towards their child despite feeling frustrated), rated on a 7-point Likert-type scale from 0 (*never*) to 6 (*very often*, e.g.,* several times a day*) at pre- and post-intervention.

### Statistical method

The primary analyses were conducted according to the intention-to-treat (ITT) principle, with all randomized participants retained in their assigned groups. Secondarily, the analyses were repeated per protocol, excluding treatment group parents not completing the intervention. For each outcome measure, a linear mixed-effects model (LMM) was run including *time* (pre, post, follow-up; treated categorically), *group* (control, treatment), and a *group by time* interaction as fixed effects, as well as a by-participant random intercept. Different covariance structures were fit in a pre-specified order, from complex to simple, until the LMM converged. The *group by time* interaction term was used to determine whether the groups differed in their trajectories of (mean) change from pre- to post- or follow-up-assessment. Effect sizes (ES) were calculated by dividing the between-group difference in model-based estimates of mean change from pre to post/follow-up by the pooled standard deviation at baseline [52, 53] and interpreted as Cohen’s *d* 0.20 = small, 0.50 = medium, and 0.80 = large [54]. To indicate the potential clinical significance of pre-to-post changes on the primary outcome measure, we post hoc calculated the proportion of participants with a reliable change on the PSE, using the Jacobson-Truax (JT) method [55, 56]. To explore the potential of IPSA in supporting families where both parent and child have ADHD, we also conducted an ancillary sensitivity analysis by repeating the primary ITT analysis, including only the subgroup of parents who had non-autistic children with ADHD. All other analyses were planned a priori. LMMs were conducted with alpha = 0.05, with no adjustments for multiplicity. Missing data were handled within the LMM, using restricted maximum likelihood-based estimations of model parameters based on all available data [57]. Model residuals were checked, detecting no extreme outliers or deviations. Analyses were performed in RStudio version 2023.06.0 + 421, using the ‘nlme’ package for LMM analyses [58] and ‘JTRCI’ to obtain and plot the JT and reliable change index indices [59].

## Results

### Study flow and participant characteristics

**Fig. 1:**
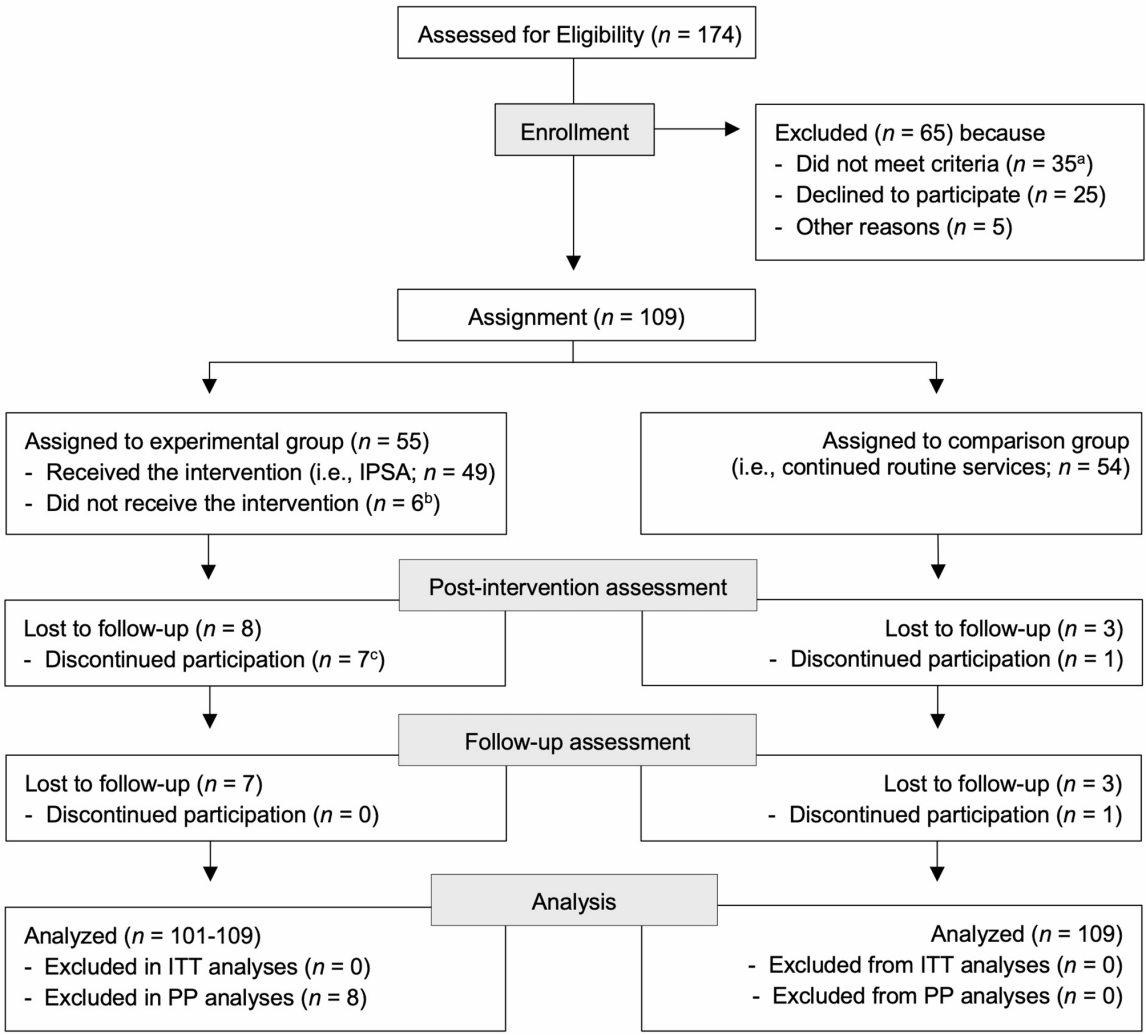
Flow of Participants Through Each Stage of the Study. *Note*. IPSA = Improving Parenting Skills Adult ADHD; ITT = intention-to-treat; PP = per protocol (excluding experimental group participants who did not complete the intervention). ^a^ e.g., diagnosed autism (*n* = 9); no child in the ages between 3 and 11 (*n* = 9); mental health or life circumstances that need to be prioritized with other types of interventions (*n* = 9), other (*n* = 8). ^b^ e.g., new work-/family-related circumstances, scheduling difficulties, could not be reached before program start. ^c^*n* = 6 of whom discontinued their participation before starting the intervention (se ^b^ for reasons)

A total of 109 parents were randomly assigned to receive IPSA in addition to their routine services (*n* = 55, 50.5%) or to continued routine services only (*n* = 54, 49.5%: Fig. 1). Forty-nine treatment group parents (89.1%) started IPSA, that is, attended at least one IPSA session. Six parents randomized to IPSA never started the program, that is, dropped out before receiving BPT, for reasons such as scheduling difficulties or changes in their work and/or family situation. Complete parent report data (primary and secondary measures) were available for 98 parents (89.9%; 47 [85.5%] treatment group parents, 51 [94.4%] comparison group parents) post intervention and for 88 parents (80.7%; 40 [72.7%] treatment group parents, 48 [88.9%] comparison group parents) at follow-up. Little’s Missing Completely At Random test was non-significant (*p* =.43).

Baseline demographic and clinical characteristics of the participants and their target children are presented in Tables 2 and 3, respectively. Most parents (86.2%) had at least one ongoing routine service or intervention (Table 2); 73.4% used ADHD medication; 35.8% used Antidepressant medication; 40.4% received some form of psychological intervention and/or psychosocial service (e.g., housing support). 71% of the parents lived together with their target child full-time, the rest lived with their child at least half time (50% or more). 34% of the target children had ADHD and/or autism and 39.5% had at least one routine service or intervention of their own (Table 3).

**Table 2:** Participant sociodemographic and clinical characteristics at baseline

	Treatment group (*n* = 55)		Control Group (*n* = 54)	
	Mean* (SD)*	Min-max	Mean* (SD)*	Min-max
Age	42.29 (5.52)	25-52	40.98 (5.53)	29-53
Years since ADHD diagnosis	3.77 (3.4)	0-12	3.40 (3.72)	0-20
Number of children	2.15 (0.78)	1-4	1.98 (0.69)	1-4
	***n***	**%**	***n***	**%**
Female gender	39	70.91	40	74.07
Education				
Secondary	19	34.55	17	31.48
University	24	43.64	28	51.85
Other^a^	12	21.82	9	16.67
Main occupation				
Working or studying^b^	43	78.18	41	75.93
Other^c^	12	21.82	13	24.07
ADHD diagnosis				
ADHD combined presentation	45	81.82	44	81.48
ADHD other presentation	10	18.18	10	18.52
≥ 1 additional psychiatric condition^d^	20	38.46	25	46.30
ADHD medication	40	72.73	40	74.07
Parallel interventions and services				
Pharmacological^e^	28	50.91	22	40.74
Pharmacological and psychological	8	14.55	11	20.37
Pharmacological and psychosocial^f^	6	10.90	9	16.67
Other^g^	5	9.09	5	9.26
No intervention	8	14.55	7	12.96
Any intervention	47	85.45	47	87.04
	**Mean (*SD*)**	**Min-max**	**Mean (*SD***)	**Min-max**
ASRS Screener score	4.24 (1.20)	2-6	4.72 (1.25)	1-6
PSE Total Scale	300.53 (52.45)	189-411	304.07 (46.92)	209-421
ECBI IS	145.02 (29.32)	71-196	148.81 (29.13)	87-221
ECBI PS	17.65 (7.08)	4-31	17.19 (6.81)	4-29

*ASRS* Adult ADHD Self-Report Scale, *ECBI IS* Eyberg Child Behavior Inventory, Intensity Scale, *ECBI PS* ECBI Problem Scale, *PSE* Parental Self-Efficacy scale

^a^e.g., primary school or vocational training

^b^figure also includes participants on parental leave (*n* = 4 in the treatment group, *n* = 2 in the control group)

^c^e.g., being on sick leave or applying for work

^d^e.g., Bipolar disorder, Depression, Anxiety or Fatigue

^e^e.g., ADHD medication, antidepressant medication, or other medication

^f^e.g., ADHD medication and housing support

^g^e.g., psychological and psychosocial intervention

**Table 3:** Demographic and clinical characteristics of the participants’ target children

	Treatment group (*n* = 55)		Control Group (*n* = 54)	
	Mean *(SD)*	Min-max	Mean *(SD)*	Min-max
Age	7.54 (2.12)	3–11	7.04 (2.29)	3–11
	***n***	**%**	***n***	**%**
Female gender	21	38.18	22	40.74
Live full time with the participating parent	40	72.73	37	68.52
NDC diagnosis^a^				
ADHD	15	27.27	12	22.22
Autism w/or w/o ADHD	5	9.09	5	9.26
Intervention/Service				
ADHD medication	10	18.18	12	22.22
Other^b^	12	21.82	9	16.67
No intervention	33	60.00	33	61.11
Any intervention	22	40.00	21	38.89

*NDC* Neurodevelopmental condition, *w/* with, *w/o* without

^a^as reported by their parent

^b^e.g., habilitation services, primary or secondary psychiatric services, municipal service

### Primary outcome

**Fig. 2:**
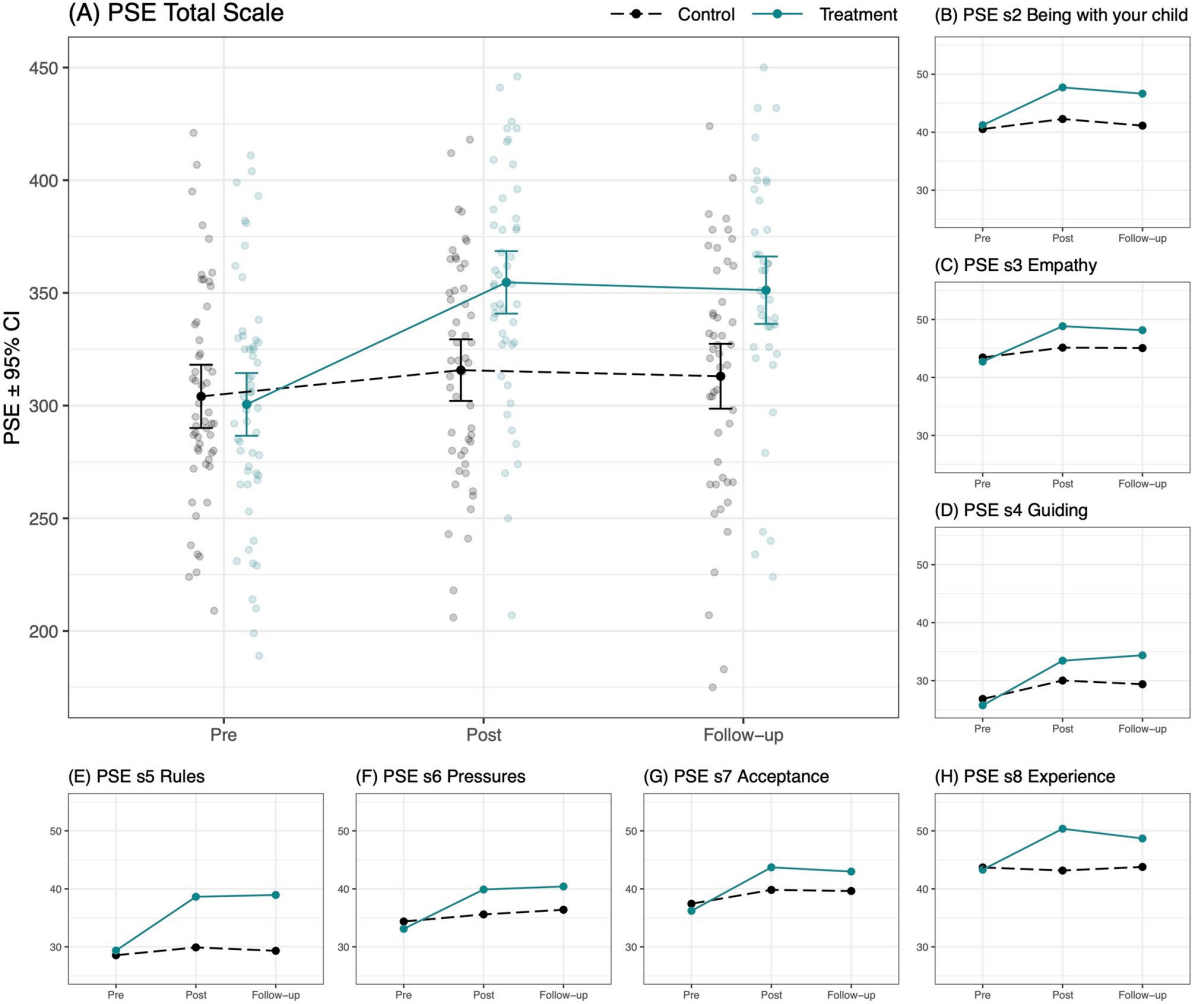
A-H. Estimated Means for the Parental Self-Efficacy Total Scale and Subscales at Pre-, Post-, and Follow-Up Assessments. Note. Plot of Parental Self Efficacy (PSE) scale scores before (Pre) and immediately after (Post) IPSA, and at Follow-up 1.5 to 3 months later, as rated by parents in the experimental/IPSA group (solid/blue line) and parents in the comparison group (dashed/black line). 95% CI = 95 % Confidence intervals. s2-s8 = subscales 2 through 8 (subscale 1 was not analyzed due to a too low Cronbach’s alpha)

A statistically significant *group by time* interaction (details in Table 4) indicated that the mean change in parental self-efficacy (PSE Total Scale) was greater for parents in the treatment group than for parents in the comparison group both immediately post IPSA (*p* <.001, *d* = 0.85) and at follow-up (*p* <.001, *d* = 0.84; Fig. 2A). The same pattern applied to the seven PSE subscales (subscales 2 through 8) whose Cronbach’s a exceeded 0.70 and thus could be analyzed (all *p* <.05, *d* between 0.42 and 0.79; Table 4; Figs. 2B-H). A re-run of the analyses per protocol did not change the results (data not shown). The number of participants with complete pre and post data (*n* = 98, 89.9%) classified as reliably improved, unchanged and deteriorated using the JT method were 27 (57.4%), 20 (42.6%) and 0 (0%) in the IPSA group and 6 (11.8%), 42 (82.4%) and 3 (5.9%) in the comparison group, respectively (Suppl. Figure 1).

**Table 4:** Results from linear Mixed-Effect model^a^ analyses of the parental Self-Efficacy total scale and subscales^b^

	Pre		Post		Follow-up		Pre to post			Pre to (1.5-3 months) follow-up		
	Treatment	Control	Treatment	Control	Treatment	Control	Between groups			Between groups		
	Mean^c^(95% CI)	Mean^c^(95% CI)	Mean^c^(95% CI)	Mean^c^(95% CI)	Mean^c^(95% CI)	Mean^c^(95% CI)	Mean change (95% CI)	*t* value (*p* value)	ES ^d^(95% CI)	Mean change (95% CI)	*t *value (*p* value)	ES ^d^(95% CI)
PSE Total Scale	300.53 (286.61, 314.44)	304.09 (290.04, 318.13)	354.68 (340.82, 368.54)	315.74 (302.07, 329.41)	351.20 (336.25, 366.15)	313.02 (298.64, 327.41)	42.50 (28.57, 56.43)	6.02 (.000)	0.85 (0.57, 1.13)	41.73 (25.89,57.58)	5.20 (.000)	0.84 (0.52, 1.16)
s2 Being with your child	41.22 (38.25, 44.19)	40.54 (37.54, 43.53)	47.72 (44.96, 50.48)	42.27 (39.57, 44.98)	46.64 (43.74, 49.55)	41.13 (38.34, 43.92)	4.76 (1.51, 8.02)	2.89 (.004)	0.44 (0.14, 0.75)	4.83 (1.42, 8.25)	2.79 (.006)	0.45 (0.13, 0.77
s3 Empathy	42.73 (40.36, 45.10)	43.44 (41.05, 45.83)	48.83 (46.52, 51.14)	45.14 (42.89, 47.39)	48.14 (45.85, 50.43)	45.06 (42.89, 47.24)	4.40 (1.47, 7.34)	2.96 (.004)	0.49 (0.16, 0.82)	3.79 (0.74, 6.85)	2.45 (.015)	0.42 (0.08, 0.76)
s4 Guiding	25.75 (23.00, 28.49)	26.84 (24.07, 29.61)	33.44 (30.41, 36.47)	30.02 (27.04, 33.00)	34.37 (31.28, 37.47)	29.38 (26.42, 32.34)	4.51 (1.39, 7.63)	2.86 (.005)	0.45 (0.14, 0.76)	6.09 (2.73, 9.44)	3.58 (.000)	0.61 (0.27, 0.94)
s5 Rules	29.36 (26.31, 32.41)	28.56 (25.48, 31.64)	38.64 (35.67, 41.61)	29.91 (26.99, 32.83)	38.95 (35.84, 42.07)	29.32 (26.31, 32.33)	7.93 (4.78, 11.07)	4.97 (.000)	0.71 (0.43, 0.99)	8.82 (5.63, 12.02)	5.45 (.000)	0.79 (0.50, 1.07)
s6 Pressures	33.11 (29.92, 36.30)	34.38 (31.15, 37.60)	39.90 (36.73, 43.06)	35.60 (32.49, 38.71)	40.42 (37.06, 43.77)	36.40 (33.20, 39.60)	5.56 (2.10, 9.02)	3.17 (.002)	0.47 (0.18, 0.76)	5.28 (1.33, 9.24)	2.64 (.009)	0.44 (0.11, 0.78)
s7 Acceptance	36.22 (33.54, 38.89)	37.44 (34.74, 40.14)	43.71 (41.14, 46.29)	39.83 (37.29, 42.36)	42.99 (40.29, 45.70)	39.63 (37.00, 42.26)	5.11 (2.47, 7.75)	3.82 (.000)	0.52 (0.25, 0.78)	4.58 (2.01, 7.15)	3.52 (.001)	0.46 (0.20, 0.72)
s8 Experience	43.31 (40.58, 46.04)	43.70 (40.94, 46.45)	50.37 (48.04, 52.71)	43.17 (40.90, 45.44)	48.69 (45.93, 51.45)	43.78 (41.17, 46.40)	7.59 (4.26, 10.92)	4.50 (.000)	0.75 (0.42, 1.07)	5.29 (1.71, 8.88)	2.91 (.004)	0.52 (0.17, 0.87)

*PSE* Parental Self Efficacy scale, *s2-s8* subscales 2 through 8

^a^Including time, group and the group by time interaction as fixed effects and a by-participant random intercept; run with covariance structures Unstructured (applies to PSE subscale 8) or Heterogenous first-order autoregressive (applies to PSE Total Scale and PSE subscales 2 through 7)

^b^Subscale 1 was not analyzed due to a too low *α *(Cronbach’s alpha, <.70)

^c^Estimated means

^d^Effect sizes for estimated pre to post/follow-up mean change and their respective confidence intervals, calculated, and interpreted as Cohen’s *d*

#### Ancillary sensitivity analysis

The results pattern did not change when the primary ITT analysis of pre-to-post change on the PSE Total Scale was exploratively repeated including only parents of children with ADHD (*n* = 27; post *p* =.002, *d* = 1.06 [95% CI 0.40, 1.72]; follow-up *p* =.012, *d* = 0.82 [95% CI 0.19, 1.45]; see Suppl. Table 3).

### Secondary outcomes

Analyses of changes in parental stress (PSS), home chaos (CHAOS), and child externalizing behaviors (ECBI IS and ECBI PS) showed greater pre-to-post intervention reductions in the treatment group than in the comparison group (all *p* <.05, *d* between 0.39 and 0.71; details in Table 5). At follow-up, the *group by time* interactions remained statistically significant for all scales except the CHAOS (details in Table 5). A re-run of analyses per protocol did not change the results (not shown).

**Table 5:** Results from linear Mixed-Effect model^a^ analyses of secondary outcome measures

	Pre		Post		Follow-up		Pre to post			Pre to (1.5-3 months) follow-up		
	Treatment	Control	Treatment	Control	Treatment	Control	Between groups			Between groups		
	Mean^b^ (95% CI)	Mean^b^ (95% CI)	Mean^b^ (95% CI)	Mean^b^ (95% CI)	Mean^b^ (95% CI)	Mean^b^ (95% CI)	Mean change (95% CI)	*t* value (*p* value)	ES ^c^ (95% CI)	Mean change (95% CI)	*t* value (*p* value)	ES^c^ (95% CI)
PSS	44.51 (41.93, 47.09)	43.94 (41.34, 46.55)	39.78 (37.00, 42.55)	43.25 (40.50, 46.01)	40.75 (38.11, 43.38)	43.73 (41.18, 46.28)	−4.04 (−6.30, −1.78)	**−**3.53 (0.001)	−0.42 (−0.65, −0.18)	−3.55 (−6.26, −0.83)	**−**2.58 (0.011)	−0.37 (−0.65, −0.09)
CHAOS	40.35 (38.34, 42.35)	43.78 (41.76, 45.80)	36.81 (34.97, 38.65)	43.23 (41.42, 45.04)	37.40 (35.46, 39.33)	42.38 (40.50, 44.26)	−2.98 (−5.02, −0.95)	**−**2.90 (0.004)	−0.42 (−0.70, −0.13)	−1.55 (−3.56, 0.47)	−1.52 (0.131)	−0.22 (−0.50, 0.06)
ECBI IS	145.02 (137.09, 152.94)	148.84 (140.84, 156.83)	130.22 (122.54, 137.91)	145.35 (137.71, 152.99)	130.89 (123.54, 138.24)	143.77 (136.53, 151.02)	−11.30 (−17.32, −5.29)	**−**3.71 (0.000)	−0.39 (−0.59, −0.18)	−9.06 (−15.10, −3.02)	**−**2.96 (0.004)	−0.31 (−0.52, −0.10)
ECBI PS	17.65 (15.71, 19.60)	17.18 (15.22, 19.14)	11.71 (9.81, 13.62)	16.21 (14.34, 18.08)	10.95 (8.99, 12.91)	15.15 (13.28, 17.03)	−4.96 (−7.16, −2.77)	**−**4.46 (0.000)	−0.71 (−1.03, −0.40)	−4.67 (−6.98, −2.37)	**−**4.00 (0.000)	−0.67 (−1.00, −0.34)

*CHAOS* Confusion, Hubbub, and Order Scale, *ECBI IS* Eyberg Child Behavior Inventory, Intensity Scale, *ECBI PS* ECBI Problem Scale, *HADS* Hospital Anxiety and Depression Scale, *PSS* Parental Stress Scale, *PSS-10* Perceived Stress Scale, 10-item version

^a^Run with covariance structure Heterogenous first-order autoregressive

^b^Estimated means

^c^Effect sizes for estimated pre to post/follow-up mean change and their respective confidence intervals, calculated and interpreted as Cohen’s *d*

### Safety/Potential harms

There were no changes regarding general perceived stress (PSS-10), anxiety (HADS Anxiety) or depression (HADS Depression), neither post IPSA (all *p* >.36), nor at follow-up (all *p* >.35). The only serious adverse event recorded occurred before the participant concerned had started IPSA and was thus judged not to be related to the intervention.

### Completion rate and use of targeted skills

Forty-seven of the 55 parents (85.5%) who were allocated to the treatment group completed the program (i.e., participated in at least nine of the 14 sessions). Among the 49 parents who started IPSA (i.e., attended at least one session), 47 (95.9%) completed the intervention. Program starters attended an average of 88.8% of sessions (median = 13, min = 5, max = 14), specifically 83.7% of group sessions (median = 6, min = 2, max = 6) and 92.6% of the individual sessions (median = 8, min = 3, max = 8).

**Fig. 3:**
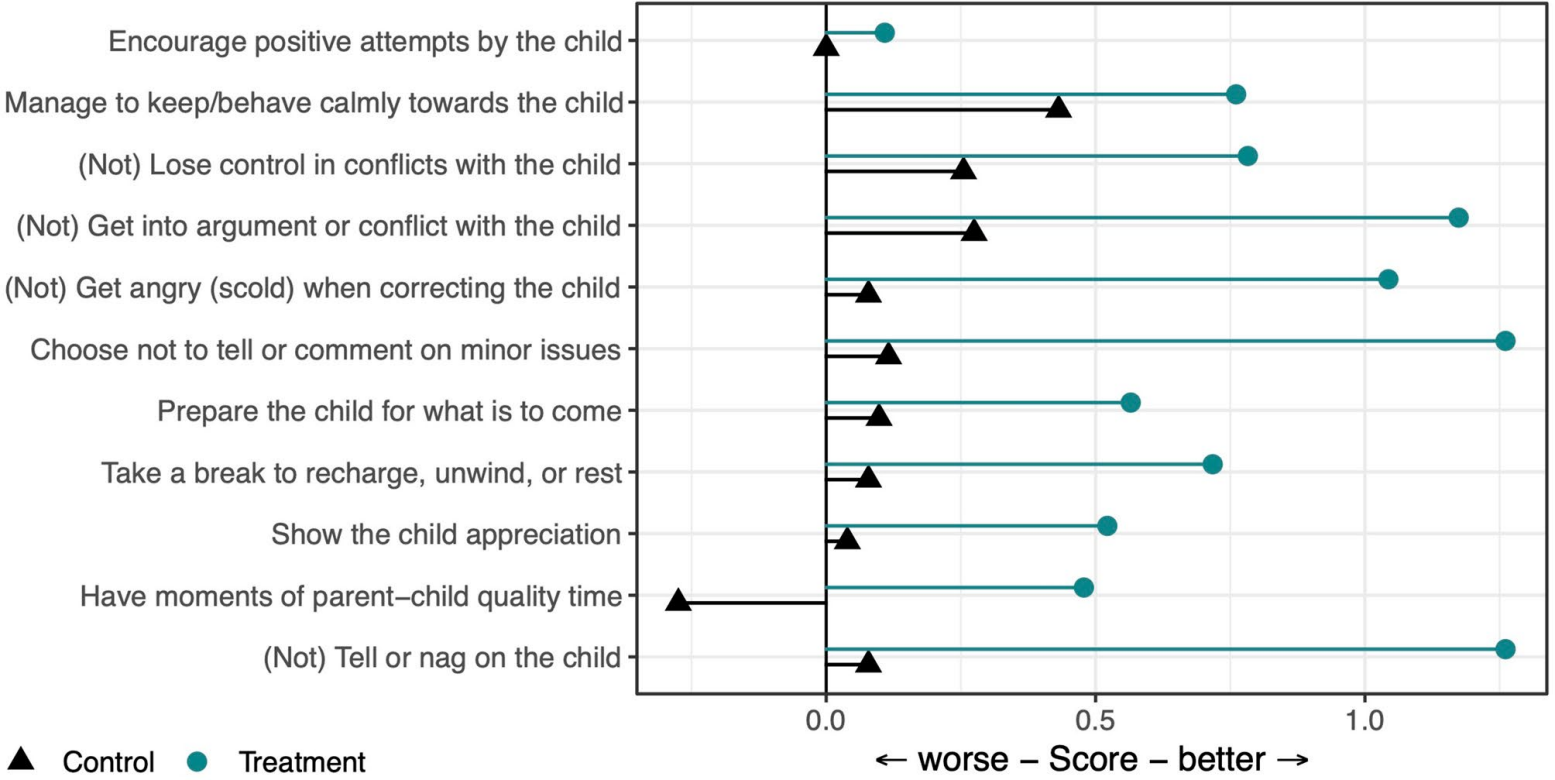
Participants’ Self-Rated Use of Targeted PT Skills and Behaviors Before and After IPSA (Mean Difference). Note. The figure shows the mean difference between parents’ pre- and post-intervention ratings (i.e., post minus pre) of how often they engaged in a set of targeted parenting behaviors, as rated on a scale from 0 (*never*) to 6 (*very often*, e.g.,* several times a day*), shown for parents in the treatment and comparison groups separately. Items relating to behaviors whose frequency should ideally decrease (marked with ‘Not’) have been reversed before insertion, so that any mean estimates lying to the right of the value zero can be interpreted as an indication of change in the desired direction

The frequency with which parents used targeted BPT skills and behaviors before and after IPSA are shown in Fig. 3, as the mean differences between their pre- and post-intervention ratings on each Skills use item. On the Skills use total scale, parents in the treatment group scored on average 35.7 (SD = 6.2) at baseline and 43.5 (SD = 6.2) post IPSA (mean pre-to-post difference = 7.8, *d* = 1.48 [95% CI = 1.05, 1.90]), while parents in the comparison group scored on average 35.3 (SD = 6.8) at baseline and 36.4 (SD = 6.2) post IPSA (mean pre-to-post difference = 1.1, *d* = 0.18 [95% CI = −0.02, 0.38]).

## Discussion

To the best of our knowledge, this is the first RCT of a BPT intervention tailored to parents with ADHD. Specifically, we examined the efficacy of a BPT program called IPSA by randomizing parents to receive IPSA alongside their routine services or to continue their routine services only, pending IPSA. We found that participation in IPSA was associated with significant improvements in parental self-efficacy. In addition, we observed a pattern of change in the expected direction across the study’s secondary outcomes, including reductions in the participants’ perceptions of externalizing behaviors in their child. Concurrently, parents in the IPSA group reported increased use of parenting skills/behaviors targeted by the intervention.

Based on previous research, we know that many parents with ADHD describe parenting-related difficulties and report low parental self-efficacy [2, 4]. Reflecting this, parents in the current sample reported considerably lower parental self-efficacy (measured by the PSE, at baseline) than that previously observed among Swedish BPT-seeking parents without known ADHD [42]. Prior research has also established that BPT can help improve parental self-efficacy [17, 18]. Consistent with this, we found a significantly greater increase in parental self-efficacy (PSE Total Scale) among parents participating in IPSA than among parents in the comparison group, with large-sized post-intervention effects surviving follow-up. Indeed, a pattern of small to medium improvements was observed across all seven PSE subscales analyzed, each intended to reflect the participants’ perceptions of their parenting within a particular parenting domain. While it is not fully understood why parents with ADHD tend to experience less favorable outcomes from conventional BPT, it has been suggested that difficulties in initiating and implementing the acquired skills at home may play a central role; an activity that puts high demands on parental executive functioning and abilities to put plans into practice [1, 22, 60]. Considering this, it is encouraging that we observed medium-sized improvements not only in self-referential parental cognitions (e.g., thoughts about being a good enough parent, able to be there for one’s child; PSE subscale Acceptance) but also regarding parents’ perceived ability to introduce changes needed to influence their child’s behavior (e.g., being able to set limits and find ways to avoid conflict; PSE subscales Rules and Experience). Indeed, among the PSE subscales, the largest changes– with medium ES at follow-up - were seen for the subscales Rules (e.g., reflecting perceptions of being able to adhere to rules and reason with one’s child), Experience (e.g., reflecting perceptions of being able to learn new ways of dealing with one’s child and handle problems using others’ advice), and Guiding (e.g., reflecting perceptions of being able to influence one’s child’s behavior and to stay/act calm in challenging situations). Concurrent reports of reduced externalizing behaviors in the participants’ target children (discussed below) may– especially if read together with reports from treatment group parents of a more frequent use of targeted PT skills and behaviors after IPSA - further indicate parental experiences of actually *doing* things in new ways at home.

In addition to improvements in parental self-efficacy, we found that parents in IPSA reported greater reductions than parents in the comparison group across the study’s secondary outcome measures, assessing parental stress (PSS), home chaos (CHAOS), and child externalizing behaviors (ECBI IS and ECBI PS). These between-group differences were small-to-medium-sized and persisted from post IPSA to follow-up for all measures except CHAOS. Regarding home chaos, the construct is not commonly assessed in BPT trials. However, it was deemed interesting to monitor, as elevated levels of home chaos have been linked to parental ADHD symptomatology, ineffective parenting, and externalizing child behaviors [8, 61]. In contrast, parents rated their children’s externalizing behaviors as less frequent (small ES) and less problematic (medium ES) both immediately after IPSA and at follow-up. This observation is reasonable, as IPSA draws on key components and skills from BPT protocols designed to reduce externalizing behaviors in children [32, 33, 62] - a type of intervention commonly associated with medium-sized effects on child behaviors [19]. But most of all, the finding is encouraging, given that previous studies of BPT involving parents with clinical-level ADHD symptomatology have shown mixed results in terms of improvements in children’s behavioral symptoms [21].

Findings on the impact of conventional BPT on parental stress and distress tend to vary– with some reporting small improvements that do not necessarily persist at follow-up [18], while others simply conclude that BPT does not worsen parental mental health (e.g., depression; [63]). In this study, we noted a small pre-to-post-intervention reduction in *parental* stress (PSS) following IPSA, while detecting no change in general stress (PSS-10). Given the bidirectional relationship between parental stress and child externalizing behaviors [64], it is not unexpected that measures of the two constructs (PSS and ECBI) changed in the same direction. Meanwhile, it is reassuring that parents did not report an increase in general stress during the study period, despite the extensive nature of the IPSA program (including 14 sessions and between-session assignments). Also, we neither detected any deterioration in parental depression or anxiety (HADS), nor any other signs of unintended harm related to program participation. As alluded to above, it is not uncommon for BPT trials to include parental depression among their secondary outcomes. In our context, however, we deemed it more appropriate to ensure that participation was not associated with any worsening of parental well-being or stress.

### Methodological considerations and limitations

As often in clinical trials of parent-mediated interventions (e.g [28, 65]), the generalizability of our results is constrained by the fact that the self-referred sample was mainly female (72%) and fairly well-educated - with an educational level comparable to that of the general population in the county of Stockholm [66], but high relative to what is commonly seen among Swedish adults with ADHD [67]. We have no information about levels of functional impairment, neither for the parents nor for their target child. However, the proportion of participants with at least one psychiatric condition in addition to their ADHD was consistent with what has been found in population-based register studies on adult ADHD [68]. Moreover, baseline ratings of child externalizing behaviors were comparable to those seen in PT trials explicitly recruiting families of children with clinical-level conduct problems (e.g [32]).

The proportion of participants who had access to at least one parallel intervention (e.g., ADHD medication) was high, but comparable between the treatment and comparison groups. It remains to be investigated how IPSA compares to conventional BPT, without ADHD adaptations.

Of note, although the trial was retrospectively registered during data collection, all data processing and analysis was carried out after the registration. All outcomes except program completion were assessed based on parental reports and parents were aware of their assigned intervention (i.e., IPSA or not) during the post BPT and follow-up assessments. For measuring the primary outcome, parental self-efficacy, self-report is the only option [69]. Nevertheless, future studies should consider involving additional informants or objective measures. Preferably, such studies should also include standardized and psychometrically evaluated measures of parenting and BPT skill utilization.

### Clinical significance and future directions

The BPT protocol put to test in this RCT is designed for parents with ADHD - a group whose clinical and cognitive profile has been associated not only with an accumulation of parenting-related challenges and negative self-referential parental cognitions, but also with less favorable outcomes from conventional evidence-based PT [1, 2, 4, 21]. In short, IPSA aims to help adults with ADHD strengthen their parenting prerequisites and skills in ways that ultimately also benefits the functioning and well-being of their family, whether or not their children have already developed ADHD traits and externalizing behaviors.

Notably, only a fourth of parents in the current study had a child with ADHD. Our sensitivity analysis suggests that IPSA was efficacious in supporting also these parents, having children with ADHD, in improving their parental self-efficacy. However, further studies are needed to shed light on the intervention’s potential to benefit multiplex families where both parent and child has ADHD - a group that is often recommended BPT as part of the child’s multimodal ADHD treatment.

By enhancing parental self-efficacy, IPSA impacts a class of parental cognitions that has implications for the adjustment and well-being of both parents and their children [11]. Among the reasons why parental self-efficacy is often targeted in interventions aimed at influencing parenting are its importance for parental mental health and its potential to mediate relationships between various child and situational factors on the one hand and parental responses on the other [11]. Also often highlighted are the theorized links between self-efficacy for a certain behavior (e.g., a skill), motivation to engage in that behavior (e.g., attempts to use that skills), goal adherence, and persistence in the face of obstacles, frustration, or risk of failure [12, 14, 17]. Relatedly, experiencing self-efficacy has been found important for parent’s motivation to put their parenting knowledge and skills into action [13]. Hopefully, the participants feel better equipped to engage in effective, albeit potentially challenging, parenting practices after IPSA, and thus to act in ways that will further promote and reinforce their sense of parenting competence.

Going forward, it needs to be investigated whether the BPT effects and high program retention observed in the current study can be found also when IPSA is delivered in a regular health care or social services context. Indeed, the attendance rate (89% of sessions) and completion rates (96%, counting parents with nine [64% of] sessions as completers) observed among parents starting IPSA compare favorably to attendance and dropout rates in many prior BPT studies [70]. However, it remains to be explored whether this was enabled by characteristics of the participating parents (e.g., fairly well-educated), facilitated by any of the ADHD adaptations made (e.g., explicitly attending to parental needs, combining group and individual sessions, or offering individualized appointment reminders), or can be explained by something else not related to program quality. Qualitative or mixed-method studies might help provide a better understanding of parents’ experiences of participating in and implementing IPSA.

Calls to make the delivery of BPT more sensitive to parental characteristics and needs (e.g., [22, 71, 72]) have previously been acknowledged for example in the development of parenting interventions for parents with intellectual disability [73] and severe mental illness [74]. Many of the accommodations made to support parents in IPSA (e.g., joint problem-solving) are based on those employed in other BPT interventions and may well be considered universal. However, IPSA also includes modifications that are likely relevant especially for adults with ADHD, such as the addition of OT support to help parents try out personalized strategies and tools to facilitate their PT skills practice and strengthen their prerequisites for managing family life both practically and relationally. In addition, the IPSA group sessions provide participants with an opportunity to exchange experiences and advice with other parents who have ADHD and face similar challenges. That said, it must be considered that IPSA is comprehensive and resource-intensive for both parents and BPT providers. The approach of drawing on the benefits of group-based BPT while accommodating ADHD-related needs for parent- and family-tailored support aligns with recommendations to increase the dose and tailor the pace of BPT; to allow repetition, target parental use of compensatory strategies, and address parental organizational and self-regulation skills [1, 21, 22, 75]. Future studies should examine if the benefits of the adaptations made outweigh the cost of the additional program components, as well as for which population of parents with ADHD IPSA may be a good fit (e.g., neither too extensive nor too demanding). Pending this, the IPSA method for tailoring BPT to adults with ADHD seems promising; a fact likely due to the active involvement of parents with ADHD throughout the program development process [31].

## Conclusion

This first RCT of the efficacy of the IPSA BPT program suggests that it has the potential to help parents with ADHD improve their parental self-efficacy, as evidenced by observations of large BPT effects both immediately after the intervention and at follow-up one and a half to three months later. Whether the study’s results - including parent-rated improvements in child externalizing behaviors and symptoms - remain stable over longer periods of time needs to be further investigated.

## Supplementary Information

Supplementary Material 1.

Supplementary Material 2.

## Data Availability

The research participants are patients at the clinical healthcare unit involved, which means that, in accordance with the ethical permit for the study, the datasets generated and analyzed in the study are not publicly available and cannot be shared.

## Abbreviations

ADHD: Attention-Deficit Hyperactivity Disorder
ASRS: Adult ADHD Self-Report Scale
CHAOS: Confusion, Hubbub, and Order Scale
ECBI IS: Eyberg Child Behavior Inventory, Intensity Scale
ECBI PS: Eyberg Child Behavior Inventory, Problem scale
HADS: Hospital Anxiety and Depression Scale
IPSA: Improving Parenting Skills Adult ADHD
OT: occupational therapist
PSE: Parental Self-Efficacy scale
PSS: Parental Stress Scale
PSS-10: Perceived Stress Scale, 10-item version
PT: parent training

## Footnotes

1 According to clinical practice and guidelines in Sweden, diagnostic ADHD assessments should follow certain basic principles (e.g., include a full medical history, differential diagnostics, and a function/activity assessment), and be carried out by an appropriately skilled team involving at least one specialist doctor and one psychologist.

## References

[1] Johnston C, Mash EJ, Miller N, Ninowski JE. Parenting in adults with attention-deficit/hyperactivity disorder (ADHD). Clin Psychol Rev. 2012;32(4):215–28. 22459785 10.1016/j.cpr.2012.01.007 PMC4838457

[2] Miklósi M, Kovács B, Janovicz J, Lelki F, Kassai R. Adult attention-deficit/hyperactivity symptoms and parental cognitions: a meta-analysis. Front Psychiatry. 2023;14. 10.3389/fpsyt.2023.1321078 PMC10807045 38268568

[3] Theule J, Wiener J, Rogers MA, Marton I. Predicting parenting stress in families of children with ADHD: parent and contextual factors. J Child Fam Stud. 2011;20(5):640–7.

[4] Park JL, Hudec KL, Johnston C. Parental ADHD symptoms and parenting behaviors: A meta-analytic review. Clin Psychol Rev. 2017;56:25–39. 28601690 10.1016/j.cpr.2017.05.003

[5] Gidziela A, Ahmadzadeh YI, Michelini G, Allegrini AG, Agnew-Blais J, Lau LY, et al. A meta-analysis of genetic effects associated with neurodevelopmental disorders and co-occurring conditions. Nat Hum Behav. 2023;7(4):642–56. 36806400 10.1038/s41562-023-01530-y PMC10129867

[6] Ronald A, de Bode N, Polderman TJC. Systematic review: how the Attention-Deficit/Hyperactivity disorder polygenic risk score adds to our Understanding of ADHD and associated traits. J Am Acad Child Adolesc Psychiatry. 2021;60(10):1234–77. 33548493 10.1016/j.jaac.2021.01.019 PMC11164195

[7] Connor DF, Steeber J, McBurnett K. A review of attention-deficit/hyperactivity disorder complicated by symptoms of oppositional defiant disorder or conduct disorder. J Dev Behav Pediatr. 2010;31(5):427–40. 20535081 10.1097/DBP.0b013e3181e121bd

[8] Mokrova I, O’Brien M, Calkins S, Keane S, Parental ADHD. Symptomology and ineffective parenting: the connecting link of home Chaos. Parenting: Sci Pract. 2010;10(2):119–35. 10.1080/15295190903212844 PMC2864040 20454604

[9] McGough JJ, Smalley SL, McCracken JT, Yang M, Del’Homme M, Lynn DE, et al. Psychiatric comorbidity in adult attention deficit hyperactivity disorder: findings from multiplex families. Am J Psychiatry. 2005;162(9):1621–7. 16135620 10.1176/appi.ajp.162.9.1621

[10] Erskine HE, Norman RE, Ferrari AJ, Chan GC, Copeland WE, Whiteford HA, et al. Long-Term outcomes of Attention-Deficit/Hyperactivity disorder and conduct disorder: A systematic review and Meta-Analysis. J Am Acad Child Adolesc Psychiatry. 2016;55(10):841–50. 27663939 10.1016/j.jaac.2016.06.016

[11] Albanese AM, Russo GR, Geller PA. The role of parental self-efficacy in parent and child well-being: A systematic review of associated outcomes. Child Care Health Dev. 2019;45(3):333–63. 30870584 10.1111/cch.12661

[12] Jones TL, Prinz RJ. Potential roles of parental self-efficacy in parent and child adjustment: a review. Clin Psychol Rev. 2005;25(3):341–63. 15792853 10.1016/j.cpr.2004.12.004

[13] Coleman PK, Karraker KH. Self-Efficacy and parenting quality: findings and future applications. Dev Rev. 1998;18(1):47–85.

[14] Maddux JE. Self-Efficacy, adaptation, and adjustment: theory, research, and application. 1st ed. New York, NY: Springer; 1995.

[15] Bandura A. Self-efficacy: toward a unifying theory of behavioral change. Psychol Rev. 1977;84(2):191–215. 847061 10.1037//0033-295x.84.2.191

[16] Newark PE, Elsässer M, Stieglitz RD, Self-Esteem. Self-Efficacy, and resources in adults with ADHD. J Atten Disord. 2016;20(3):279–90. 23074301 10.1177/1087054712459561

[17] Wittkowski A, Dowling H, Smith DM. Does engaging in a Group-Based intervention increase parental Self-efficacy in parents of preschool children?? A systematic review of the current literature. J Child Fam Stud. 2016;25(11):3173–91. 27795657 10.1007/s10826-016-0464-z PMC5061830

[18] Weber L, Kamp-Becker I, Christiansen H, Mingebach T. Treatment of child externalizing behavior problems: a comprehensive review and meta-meta-analysis on effects of parent-based interventions on parental characteristics. Eur Child Adolesc Psychiatry. 2019;28(8):1025–36. 29948228 10.1007/s00787-018-1175-3

[19] Mingebach T, Kamp-Becker I, Christiansen H, Weber L. Meta-meta-analysis on the effectiveness of parent-based interventions for the treatment of child externalizing behavior problems. PLoS ONE. 2018;13(9). 10.1371/journal.pone.0202855 PMC6157840 30256794

[20] Tarver J, Daley D, Sayal K. Beyond symptom control for attention-deficit hyperactivity disorder (ADHD): what can parents do to improve outcomes? Child Care Health Dev. 2015;41(1):1–14. 24910021 10.1111/cch.12159

[21] Chronis-Tuscano A, Wang CH, Woods KE, Strickland J, Stein MA. Parent ADHD and Evidence-Based treatment for their children: review and directions for future research. J Abnorm Child Psychol. 2017;45(3):501–17. 28025755 10.1007/s10802-016-0238-5 PMC5357146

[22] Crandall A, Deater-Deckard K, Riley AW. Maternal emotion and cognitive control capacities and parenting: A conceptual framework. Dev Rev. 2015;36:105–26. 26028796 10.1016/j.dr.2015.01.004 PMC4445866

[23] Assemany AE, McIntosh DE. Negative treatment outcomes of behavioral parent training programs. Psychol Sch. 2002;39(2):209–19.

[24] Reyno SM, McGrath PJ. Predictors of parent training efficacy for child externalizing behavior problems–a meta-analytic review. J Child Psychol Psychiatry. 2006;47(1):99–111. 16405646 10.1111/j.1469-7610.2005.01544.x

[25] Kazdin AE, Holland L, Crowley M. Family experience of barriers to treatment and premature termination from child therapy. J Consult Clin Psychol. 1997;65(3):453–63. 9170769 10.1037//0022-006x.65.3.453

[26] Chronis-Tuscano A, French W, Strickland J, Sasser T, Gonzalez ENS, Whitlock KB et al. Acute Effects of Parent Stimulant Medication Versus Behavioral Parent Training on Mothers’ ADHD, Parenting Behavior, and At-Risk Children. J Clin Psychiatry. 2020;81(5). 10.4088/JCP.19m13173 32926603

[27] Schoenfelder EN, Chronis-Tuscano A, Strickland J, Almirall D, Stein MA. Piloting a sequential, multiple assignment, randomized trial for mothers with Attention-Deficit/Hyperactivity disorder and their At-Risk young children. J Child Adolesc Psychopharmacol. 2019;29(4):256–67. 30950637 10.1089/cap.2018.0136 PMC6534090

[28] Jans T, Jacob C, Warnke A, Zwanzger U, Groß-Lesch S, Matthies S, et al. Does intensive multimodal treatment for maternal ADHD improve the efficacy of parent training for children with ADHD? A randomized controlled multicenter trial. J Child Psychol Psychiatry. 2015;56(12):1298–313. 26123832 10.1111/jcpp.12443

[29] Häge A, Alm B, Banaschewski T, Becker K, Colla M, Freitag C, et al. Does the efficacy of parent-child training depend on maternal symptom improvement? Results from a randomized controlled trial on children and mothers both affected by attention-deficit/hyperactivity disorder (ADHD). Eur Child Adolesc Psychiatry. 2018;27(8):1011–21. 29362929 10.1007/s00787-018-1109-0

[30] Babinski DE, Waxmonsky JG, Pelham WE. Jr. Treating parents with attention-deficit/hyperactivity disorder: the effects of behavioral parent training and acute stimulant medication treatment on parent-child interactions. J Abnorm Child Psychol. 2014;42(7):1129–40. 24687848 10.1007/s10802-014-9864-y

[31] Lindström T, Buddgård S, Westholm L, Forster M, Bölte S, Hirvikoski T. Parent training tailored to parents with ADHD: development of the improving parenting skills adult ADHD (IPSA) program. J Atten Disord. 2024;28(4):531–41. 38152999 10.1177/10870547231217090 PMC10838472

[32] Kling A, Forster M, Sundell K, Melin L. A randomized controlled effectiveness trial of parent management training with varying degrees of therapist support. Behav Ther. 2010;41(4):530–42. 21035616 10.1016/j.beth.2010.02.004

[33] Barkley R. Defiant children: a clinician’s manual for assessment and parent training. 2nd ed. New York; London: Guilford Press; 1997.

[34] Adamou M, Asherson P, Arif M, Buckenham L, Cubbin S, Dancza K et al. Recommendations for occupational therapy interventions for adults with ADHD: a consensus statement from the UK adult ADHD network. BMC Psychiatry. 2021;21. 10.1186/s12888-021-03070-z PMC7863422 33541313

[35] Hoffmann TC, Glasziou PP, Boutron I, Milne R, Perera R, Moher D, et al. Better reporting of interventions: template for intervention description and replication (TIDieR) checklist and guide. BMJ. 2014;348:g1687. 24609605 10.1136/bmj.g1687

[36] Butcher NJ, Monsour A, Mew EJ, Chan AW, Moher D, Mayo-Wilson E, et al. Guidelines for reporting outcomes in trial reports: the CONSORT-Outcomes 2022 extension. JAMA. 2022;328(22):2252–64. 36511921 10.1001/jama.2022.21022

[37] Barber JP, Liese BS, Abrams MJ. Development of the cognitive therapy adherence and competence scale. Psychother Res. 2003;13(2):205–21.

[38] Sanders MR, Spry CS, Tellegen CL, Kirby JN, Metzler CM, Prinz RJ. Development and validation of fidelity monitoring and enhancement in an evidence-based parenting program. J Behav Health Serv Res. 2020;47(4):569–80. 32476093 10.1007/s11414-020-09713-5

[39] Bywater T, Gridley N, Berry V, Blower S, Tobin K. The parent programme implementation checklist (PPIC): the development and testing of an objective measure of skills and fidelity for the delivery of parent programmes. Child Care Pract. 2019;25(3):281–309.

[40] Kessler RC, Adler L, Ames M, Demler O, Faraone S, Hiripi E, et al. The world health organization adult ADHD Self-Report scale (ASRS): a short screening scale for use in the general population. Psychol Med. 2005;35(2):245–56. 15841682 10.1017/s0033291704002892

[41] Kendall S, Bloomfield L. Developing and validating a tool to measure parenting self-efficacy. J Adv Nurs. 2005;51(2):174–81. 15963189 10.1111/j.1365-2648.2005.03479.x

[42] Ulfsdotter M, Enebrink P, Lindberg L. Effectiveness of a universal health-promoting parenting program: a randomized waitlist-controlled trial of all children in focus. BMC Public Health. 2014;14:1083. 10.1186/1471-2458-14-1083 PMC4210619 25326710

[43] Berry JO, Jones WH. The parental stress Scale - Initial psychometric evidence. J Social Personal Relationships. 1995;12(3):463–72.

[44] Lindström T, Holmberg Bergman T, Annerstedt M, Forster M, Bölte S, Hirvikoski T. Psychometric properties of the parental stress scale in Swedish parents of children with and without neurodevelopmental conditions. Scandinavian J Child Adolesc Psychiatry Psychol. 2024;12(1):10–22. 10.2478/sjcapp-2024-0002 PMC11027036 38645569

[45] Matheny AP, Wachs TD, Ludwig JL, Phillips K. Bringing order out of chaos: psychometric characteristics of the confusion, hubbub, and order scale. J Appl Dev Psychol. 1995;16(3):429–44.

[46] Eyberg SM, Ross AW. Assessment of child behavior problems: the validation of a new inventory. J Clin Child Psychol. 1978;7(2):113–6.

[47] Axberg U, Johansson Hanse J, Broberg AG. Parents’ description of conduct problems in their children - a test of the Eyberg child behavior inventory (ECBI) in a Swedish sample aged 3–10. Scand J Psychol. 2008;49(6):497–505. 18705675 10.1111/j.1467-9450.2008.00670.x

[48] Cohen S, Kamarck T, Mermelstein R. A global measure of perceived stress. J Health Social Behav. 1983;24(4):385–96. 6668417

[49] Nordin M, Nordin S. Psychometric evaluation and normative data of the Swedish version of the 10-item perceived stress scale. Scand J Psychol. 2013;54(6):502–7. 24118069 10.1111/sjop.12071

[50] Zigmond AS, Snaith RP. The hospital anxiety and depression scale. Acta Psychiatrica Scandinavia. 1983;67(6):361–70. 10.1111/j.1600-0447.1983.tb09716.x 6880820

[51] Lisspers J, Nygren A, Söderman E. Hospital anxiety and depression scale (HAD): some psychometric data for a Swedish sample. Acta Psychiatrica Scandinavica. 1997;96(4):281–6. 9350957 10.1111/j.1600-0447.1997.tb10164.x

[52] Feingold A. Confidence interval Estimation for standardized effect sizes in multilevel and latent growth modeling. J Consult Clin Psychol. 2015;83(1):157–68. 25181028 10.1037/a0037721 PMC4324017

[53] Feingold A. Effect sizes for growth-modeling analysis for controlled clinical trials in the same metric as for classical analysis. Psychol Methods. 2009;14(1):43–53. 19271847 10.1037/a0014699 PMC2712654

[54] Cohen J. Statistical power analysis for the behavioral sciences. 2nd ed. Hillsdale: Lawrence Erlbaum Associates; 1988.

[55] Blampied NM. Reliable change and the reliable change index: Still useful after all these years? the Cognitive Behaviour Therapist. 2022;15.

[56] Jacobson NS, Roberts LJ, Berns SB, McGlinchey JB. Methods for defining and determining the clinical significance of treatment effects: description, application, and alternatives. J Consult Clin Psychol. 1999;67(3):300–7. 10369050 10.1037//0022-006x.67.3.300

[57] Krueger C, Tian L. A comparison of the general linear mixed model and repeated measures ANOVA using a dataset with multiple missing data points. Biol Res Nurs. 2004;6(2):151–7. 15388912 10.1177/1099800404267682

[58] Pinheiro J, Bates D, Team RC. nlme: Linear and Nonlinear Mixed Effects Models. R package version 3.1–164. 2023.

[59] Kruijt A. _JTRCI: obtain and plot Jacobson-Truax and reliable change indices_. R package version 0.1.0. 2024. https://github.com/AWKruijt/JT-RCI.

[60] Friedman LM, Dvorsky MR, McBurnett K, Pfiffner LJ. Do parents’ ADHD symptoms affect treatment for their children?? The impact of parental ADHD on adherence to behavioral parent training for childhood ADHD. J Abnorm Child Psychol. 2020;48(11):1425–37. 32813210 10.1007/s10802-020-00672-1 PMC7567125

[61] Marsh S, Dobson R, Maddison R. The relationship between household chaos and child, parent, and family outcomes: a systematic scoping review. BMC Public Health. 2020;20(1):513. 10.1186/s12889-020-08587-8 PMC7175577 32316937

[62] Daley D, Van Der Oord S, Ferrin M, Cortese S, Danckaerts M, Doepfner M, et al. Practitioner review: current best practice in the use of parent training and other behavioural interventions in the treatment of children and adolescents with attention deficit hyperactivity disorder. J Child Psychol Psychiatry. 2018;59(9):932–47. 29083042 10.1111/jcpp.12825

[63] Waldrop J, Baker M, Salomon R, Moreton E. Parenting interventions and secondary outcomes related to maternal mental health: A systematic review. Matern Child Health J. 2021;25(6):870–80. 33905064 10.1007/s10995-021-03130-6 PMC10916505

[64] Barroso NE, Mendez L, Graziano PA, Bagner DM. Parenting stress through the Lens of different clinical groups: a systematic review & Meta-Analysis. J Abnorm Child Psychol. 2018;46(3):449–61. 28555335 10.1007/s10802-017-0313-6 PMC5725271

[65] Fabiano GA. Father participation in behavioral parent training for ADHD: review and recommendations for increasing inclusion and engagement. J Fam Psychol. 2007;21(4):683–93. 18179340 10.1037/0893-3200.21.4.683

[66] Statistics Sweden S. Educational attainment of the population 2022. 2022.

[67] Garcia-Argibay M, Pandya E, Ahnemark E, Werner-Kiechle T, Andersson LM, Larsson H, et al. Healthcare utilization and costs of psychiatric and somatic comorbidities associated with newly diagnosed adult ADHD. Acta Psychiatrica Scandinavica. 2021;144(1):50–9. 33749845 10.1111/acps.13297

[68] Giacobini M, Ahnemark E, Medin E, Freilich J, Andersson M, Ma Y, et al. Epidemiology, treatment patterns, comorbidities, and concomitant medication in patients with ADHD in sweden: A Registry-Based study (2018–2021). J Atten Disord. 2023;27(12):1309–21. 37282510 10.1177/10870547231177221

[69] Vance AJ, Brandon DH. Delineating among parenting confidence, parenting Self-Efficacy, and competence. Adv Nurs Sci. 2017;40(4):E18–37. 10.1097/ANS.0000000000000179 PMC5664183 28825934

[70] Chacko A, Jensen SA, Lowry LS, Cornwell M, Chimklis A, Chan E, et al. Engagement in behavioral parent training: review of the literature and implications for practice. Clin Child Fam Psychol Rev. 2016;19(3):204–15. 27311693 10.1007/s10567-016-0205-2

[71] Law J, Plunkett C, Taylor J, Gunning M. Developing policy in the provision of parenting programmes: integrating a review of reviews with the perspectives of both parents and professionals. Child Care Health Dev. 2009;35(3):302–12. 19250254 10.1111/j.1365-2214.2009.00939.x

[72] Smith E, Koerting J, Latter S, Knowles MM, McCann DC, Thompson M, et al. Overcoming barriers to effective early parenting interventions for attention-deficit hyperactivity disorder (ADHD): parent and practitioner views. Child Care Health Dev. 2015;41(1):93–102. 24814640 10.1111/cch.12146 PMC4283979

[73] Glazemakers I, Deboutte D. Modifying the ‘positive parenting program’ for parents with intellectual disabilities. J Intellect Disabil Res. 2013;57(7):616–26. 22554356 10.1111/j.1365-2788.2012.01566.x

[74] Radley J, Sivarajah N, Moltrecht B, Klampe ML, Hudson F, Delahay R, et al. A scoping review of interventions designed to support parents with mental illness that would be appropriate for parents with psychosis. Front Psychiatry. 2021;12:787166. 35153857 10.3389/fpsyt.2021.787166 PMC8828543

[75] Thompson MJ, Laver-Bradbury C, Ayres M, Le Poidevin E, Mead S, Dodds C, et al. A small-scale randomized controlled trial of the revised new forest parenting programme for preschoolers with attention deficit hyperactivity disorder. Eur Child Adolesc Psychiatry. 2009;18(10):605–16. 19404717 10.1007/s00787-009-0020-0

[76] Lindström T. Parents with ADHD: parenting, stress, and interventions. Karolinska Institutet. 2024.

