# Supplementary material for: Parent training tailored for parents with ADHD: a randomized controlled trial
Source: BMC Psychiatry. 2025 Aug 26;25:818. doi: 10.1186/s12888-025-07166-8 (PMC12379403; doi:10.1186/s12888-025-07166-8)
Supplement: Supplementary file 1 — Supplementary Material 1. [file 12888_2025_7166_MOESM1_ESM.pdf]

Supplementary material to

Parent training tailored for parents with ADHD: A randomized controlled trial

Content

Suppl. Table 1. Telephone screening interview questions translated into English

Suppl. Table 2. Outline of sessions in the *Improving Parenting Skills Adult ADHD* (IPSA) program

Suppl. Figure 1. Plot of Reliable Change in Parental Self-Efficacy From Pre to Post IPSA

Suppl. Table 3. Linear Mixed-Effect Model Ancillary Sensitivity Analysis of the Parental Self-Efficacy Total Scale, including only multiplex families

Supplemental Table 1. Telephone screening interview questions translated into English

**Introduction**<br>Give information about IPSA, the study, study participation, etc.		
1. Is the parent still interested in participating?	☐ **No**	☐ **Yes**
How did the parent receive information about IPSA? Specify:		

If yes to question 1, continue forward

**Screening**<br>Explain that you will ask a number of questions to see if the parent meets the study eligibility criteria (currently a prerequisite for participation)		
2. Does the parent have an ADHD diagnosis?	☐ **No**	☐ **Yes**
If yes: 3. Does the parent have an autism spectrum diagnosis?<br>(IPSA is adapted to ADHD; it is not possible to participate if you have ASD)	☐ **Yes**	☐ **No**
If no: 4. Does the parent have an intellectual disability diagnosis?<br>(same as above)	☐ **Yes**	☐ **No**
If no: 5. Does the parent have a child between 3 and 11 years old<br>who will be three years old in [.….] but will not be 12 years old by [……..] and with whom the parent wants to strengthen the relationship, increase cooperation and reduce conflict?<br>(during IPSA, the work is mainly focused on one child)	☐ **No**<br>Age of child:	☐ **Yes**
If yes: 6a. What percentage of time does the child live with the parent?	% of time:	
6b. Does the child live with the parent at least every two weeks [half time] or equivalent? (must be regular)	☐ **No**	☐ **Yes**
If yes: 7. Would the parent be able to participate in the whole IPSA program regardless of which of the groups he/she is assigned to (in […] or in […])?<br>(6 group sessions every 2 weeks + individual sessions in the weeks in between)	☐ **No**	☐ **Yes**
If yes: 8. Can the parent participate without the help of an interpreter?	☐ **No**	☐ **Yes**
If yes: 9. When was the parent diagnosed with ADHD? Specify year:		
10. Where did the parent do their clinical ADHD assessment? Specify:		

If no: 11a. Does the parent have any psychiatric condition or diagnosis in addition to their ADHD? E.g., fatigue, anxiety, depression, bipolar, similar?<br><br>If yes, specify which diagnosis(es)/condition(s):<br><br><br>If yes, explain that you need to know a bit more about the parent's psychological state/worries in order to know if IPSA is the right intervention right now - or if there is something more urgent, that needs to be prioritized over IPSA.<br><br>- Examine the severity of the parent's psychiatric co-morbidity. How much impact does it have on well-being, functioning, needs, etc.?<br>- Does the parent have any ongoing treatments/interventions?<br>If yes, state what and where:<br>- Does the parent know where to turn if they feel worse?<br>- Is the parent able/able to attend IPSA now, given the current situation?	☐ Yes	☐ No
12. Make an assessment: Does the parent have any serious psychiatric condition that needs to be treated and prioritized over IPSA? E.g., severe depression, suicidal thoughts, psychotic illness, substance abuse, etc.?<br><br>If yes, state the condition and what recommendation is given:	☐ **Yes**	☐ **No**
13. Make an assessment: Is there any treatment, contact or intervention the parent should prioritize now, over IPSA?<br><br>If yes, indicate which intervention and what recommendation is given:	☐ **Yes**	☐ **No**
If no: 14. What is the situation in the family? Is there anything in particular, anything critical that would need to be addressed and prioritized, over IPSA? E.g., a major change, e.g., a move; a major concern, e.g., serious illness; a major uncertainty, e.g., regarding finances or housing; some kind of crisis?<br><br>If yes, state what and what recommendation is given:	☐ **Yes**	☐ **No**
If no: 15. Does the child aged 3-11 that the parent would like to focus on during IPSA have any diagnosis, conditions, challenges or special needs? E.g.,: ADHD, ASD, ID, oppositional defiance disorder, conduct disorder, depression, worry, anxiety, other special needs?<br><br>If yes, specify which diagnosis, what needs:<br><br><br>Examine the overall impact of the child's needs on family daily life<br>(in order to decide whether IPSA is the right intervention at this time):	☐ No	☐ Yes

16. Does the child (same as above) have any interventions or treatments? If not, is the child currently in need of any intervention or treatment of their own?<br>If yes, specify what:<br><br>Explain that IPSA PT strategies are general and can help make a difference but will be not enough for all families, so you need to consider whether the child/family need other support/interventions at the same time or instead.	☐ No	☐ Yes
17. Make an assessment: Is there any child or family treatment, contact or intervention that should be prioritized now, over IPSA?<br>If yes, indicate what and what recommendation is given:	☐ **Yes**	☐ **No**
If no: 18. Is the parent able to participate in the program as described below?<br>[Information about program procedures, dates and time for group sessions, etc.]	☐ **No**	☐ **Yes**

**Summary assessment**		
Does the parent seem to meet all eligibility criteria?	☐ **No**	☐ **Yes**
**If no**: Give reason for exclusion at this stage:		

If yes, provide more information about study procedures, the next step, etc.

18. Does the parent agree to the above [i.e., summary of study procedures etc.]? Does the parent agree to make an appointment for the next step?	☐ **No**	☐ **Yes**

If yes, provide more practical information about the next step, make an appointment the clinic, etc.

Suppl. Table 2. Outline of sessions in the *Improving Parenting Skills Adult ADHD* (IPSA) program

Session (length)	Focus	Therapist(s)
Individual intake session (90)	Introduction. Assessment. Goal formulation. Planning.	Both
OT session (60)	Individualized	OT
Group session 1 (150)	Parental ADHD in everyday family life	Both
OT session (60)	Individualized	OT
Group session 2 (150)	How parents can strengthen their own prerequisites for managing challenging parent-child interaction situations	Both
OT session (60)	Individualized	OT
Group session 3 (150)	How parents can use positive reinforcement and strengthen the parent-child relationship through active/positive time together	Both
OT session (60)	Individualized	OT
Group session 4 (150)	How parents can facilitate parent-child cooperation e.g., by preparing transitions and communicating effectively	Both
OT session (60)	Individualized	OT
Group session 5 (150)	How parents can use strategies to regulate their own emotional expressions and reduce the risk of emotional escalation	Both
OT session (60)	Individualized	OT
Group session 6 (150)	How parents can increase predictability and reduce the risk of parent-child conflict, e.g., by choosing their battles wisely	Both
Individual closing session (60)	Summary. Assessment. Planning ahead, for maintenance.	Both
Booster group session (150)	Repetition. Booster.	Both

Note. OT = occupational therapist. Both = both occupational therapist and clinical psychologist

Supplemental Figure 1. Plot of Reliable Change * in Parental Self-Efficacy From Pre to Post IPSA, as Measured with the Parental Self-Efficacy Total Scale (Primary Outcome; n = 98)

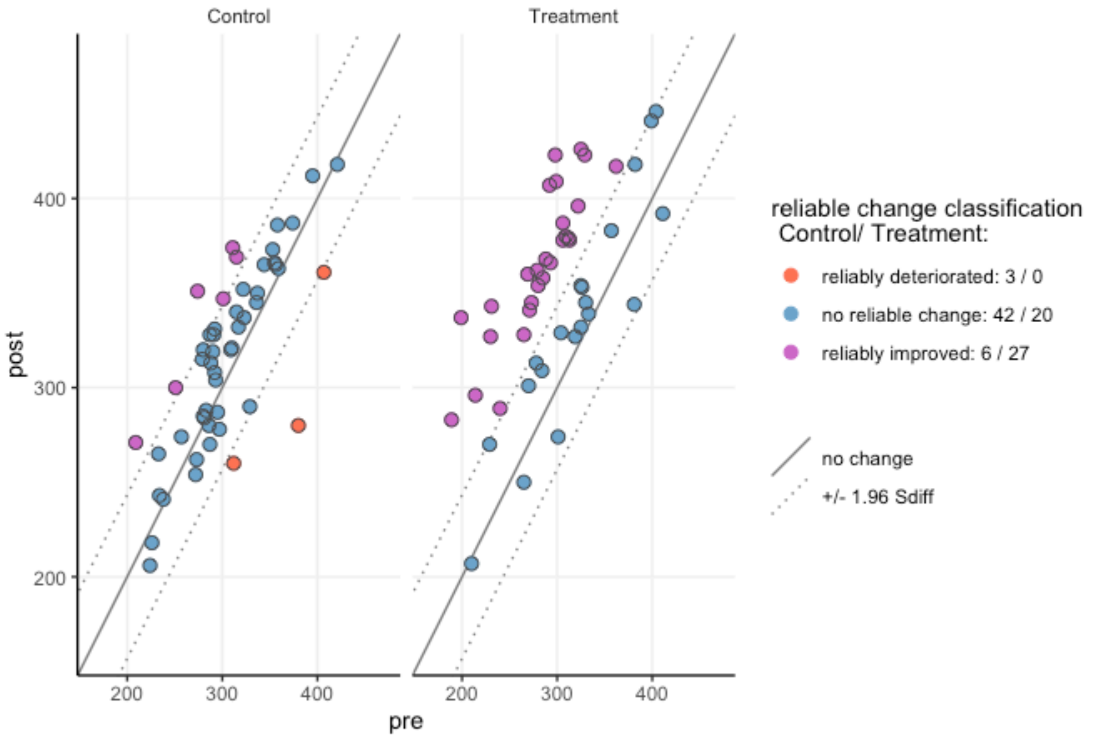

* Assuming that higher scores are better (increase = improvement)

Table 3. Linear Mixed-Effect Model [a] Ancillary Sensitivity Analysis of the Parental Self-Efficacy Total Scale, including only multiplex families

	**Pre**		**Post**		**Follow-up**		**Pre to post**			**Pre to (1.5-3 months) follow-up**		
	**Treatment**	**Control**	**Treatment**	**Control**	**Treatment**	**Control**	**Between groups**			**Between groups**		
	Mean [c] (95% CI)	Mean [c] (95% CI)	Mean [c] (95% CI)	Mean [c] (95% CI)	Mean [c] (95% CI)	Mean [c] (95% CI)	Mean change (95% CI)	*t* value (*p* value)	ES [d] (95% CI)	Mean change (95% CI)	*t* value (*p* value)	ES [d] (95% CI)
PSE Total Scale	305.80 (280.08, 331.52)	288.99 (260.35, 317.63)	353.26 (329.10, 377.41)	291.43 (264.23, 318.63)	345.69 (319.10, 372.28)	294.15 (264.18, 324.13)	45.02 (17.01, 73.03)	**3.25** (.002)	1.06 (0.40, 1.72)	34.73 (8.11, 61.35)	**2.64** (.012)	0.82 (0.19, 1.45)

Note. CI = Confidence Interval; PSE = Parental Self Efficacy scale

[a] Including time, group and the group by time interaction as fixed effects and a by-participant random intercept; run with covariance structure Heterogenous first-order autoregressive

[c] Estimated means // [d] Effect sizes for estimated pre to post/follow-up mean change and their respective confidence intervals, calculated, and interpreted as Cohen's
